# Women’s experiences of a diagnosis of gestational diabetes mellitus: a systematic review

**DOI:** 10.1186/s12884-020-2745-1

**Published:** 2020-02-07

**Authors:** Louise Craig, Rebecca Sims, Paul Glasziou, Rae Thomas

**Affiliations:** Institute for Evidence-Based Healthcare, Bond University, Gold Coast, Australia

**Keywords:** Gestational diabetes mellitus, Systematic review, Qualitative, Diagnostic impacts

## Abstract

**Background:**

Gestational diabetes mellitus (GDM) - a transitory form of diabetes induced by pregnancy - has potentially important short and long-term health consequences for both the mother and her baby. There is no globally agreed definition of GDM, but definition changes have increased the incidence in some countries in recent years, with some research suggesting minimal clinical improvement in outcomes. The aim of this qualitative systematic review was to identify the psychosocial experiences a diagnosis of GDM has on women during pregnancy and the postpartum period.

**Methods:**

We searched CINAHL, EMBASE, MEDLINE and PsycINFO databases for studies that provided qualitative data on the psychosocial experiences of a diagnosis of GDM on women across any stage of pregnancy and/or the postpartum period. We appraised the methodological quality of the included studies using the Critical Appraisal Skills Programme Checklist for Qualitative Studies and used thematic analysis to synthesis the data.

**Results:**

Of 840 studies identified, 41 studies of diverse populations met the selection criteria. The synthesis revealed eight key themes: initial psychological impact; communicating the diagnosis; knowledge of GDM; risk perception; management of GDM; burden of GDM; social support; and gaining control. The identified benefits of a GDM diagnosis were largely behavioural and included an opportunity to make healthy eating changes. The identified harms were emotional, financial and cultural. Women commented about the added responsibility (eating regimens, appointments), financial constraints (expensive food, medical bills) and conflicts with their cultural practices (alternative eating, lack of information about traditional food). Some women reported living in fear of risking the health of their baby and conducted extreme behaviours such as purging and starving themselves.

**Conclusion:**

A diagnosis of GDM has wide reaching consequences that are common to a diverse group of women. Threshold cut-offs for blood glucose levels have been determined using the risk of physiological harms to mother and baby. It may also be advantageous to consider the harms and benefits from a psychosocial and a physiological perspective. This may avoid unnecessary burden to an already vulnerable population.

## Background

Gestational diabetes mellitus (GDM) is diagnosed by elevated blood glucose in pregnancy though the definition has changed repeatedly since its first description in the 1960’s [1, 2]. The most frequently reported perinatal consequence of GDM is macrosomia (usually defined as a neonate weighing over 4 kg) which can increase the risk of caesarean section and shoulder dystocia. For the mother, there are also potential longer-term consequences including an increased risk of type 2 diabetes post-pregnancy and/or in later life [3]. The investigators of a large international Hyperglycemia and Adverse Pregnancy Outcome (HAPO) study aimed to identify a cut-point in the continuum to decide the blood glucose level (BGL) thresholds that should be used to define GDM [4]. However, a definitive cut-point was not identified and using the HAPO data the International Association of the Diabetes and Pregnancy Study Groups (IADSPG) consensus panel recommended a BGL threshold associated with the risk of adverse infant outcomes (such as risk of macrosomia, excess infant adiposity and neonatal hyperinsulinemia) [5]. This change was controversial, and there is currently a lack of an agreed standard for diagnosing high blood glucose in pregnancy.

Pregnancy can be a vulnerable period when a woman is adapting and responding to changes in body perceptions, such as loss of strength or fitness, which can result in reduced self-esteem and depression [6]. Many women report depression and anxiety during pregnancy which often includes worry for the baby’s wellbeing [7, 8]. A diagnosis of a health condition such as GDM could have a detrimental effect on a pregnant woman’s quality of life due to fears that the illness may affect her and/or her baby [9]. This has potential to convert pregnancy, a natural process, into one associated with risks, ill-health and increased surveillance [10]. Understanding a women’s response to the GDM diagnosis and its psychological impact has emerged as an important issue [11]. Some studies report women describing the initial response as one of ‘shock’ [12, 13], ‘sadness’ and ‘guilt’ [13]. A women’s acceptance of risk and fear of complications is likely to influence the acceptability of various interventions. Therefore, it is imperative to amalgamate the findings of these studies to synthesise the array of potential psychosocial consequences of a diagnosis of GDM.

In many countries the prevalence of GDM is rising [14–16]. Some of this is due to the increasing age at which women are becoming pregnant, an increase in obesity amongst women, more testing during pregnancy, and better recording during pregnancy. However, much of the rise has occurred since 2013 when some countries adopted the new IADPSG criteria and testing regimen for gestational diabetes. This resulted in the anomalous position that two women in two countries with exactly the same glucose levels may or may not be diagnosed with GDM depending on the country’s definition. Caution had been previously raised that the new IADPSG definition would increase prevalence of women diagnosed with GDM by two-to-three-fold [17].

Despite a significant increase in prevalence of GDM after the introduction of the new IADPSG criteria [15, 16], some pre-post studies suggest negligible clinical improvement in the adverse outcomes measured [17, 18]. Findings from a qualitative study of 19 women of different cultural backgrounds investigating women’s experiences of a GDM diagnosis reported that the diagnostic criteria itself was viewed as ‘confusing’ by some women and treatment for their ‘borderline’ condition unnecessary [19].

Although multiple studies have considered the impact of a diagnosis of GDM, a systematic review to synthesise the evidence around the emotional impact of a diagnosis at different stages, i.e. time of diagnosis, after diagnosis, at the delivery of the baby, and post-delivery, is lacking. The findings could inform healthcare clinicians of women’s attitudes and the consequences of a diagnosis and illuminate potential opportunities to provide support and advise. Therefore, in this systematic review, we aim to synthesise the evidence of the psychosocial experiences a diagnosis of GDM has on women during pregnancy and the postpartum period.

## Methods

We followed the Enhancing Transparency in Reporting the Synthesis of Qualitative Research Guidelines (ENTREQ; Additional file 1: Table S1) [20]. We included primary studies published in peer-review journals that:

- included pregnant women with a current diagnosis or women with a history of GDM;
- provided qualitative data on the psychosocial experiences of a diagnosis of GDM on women across any stage of pregnancy and/or the postpartum period; *and*
- where participants have provided an account of their experience or perspective of living with GDM

No restrictions were placed on country, written language, or year of publication.

Studies were excluded, if:

- the primary aim was to identify barriers and/or facilitators to service as these focused on the management of GDM rather than the GDM diagnosis; or
- participants were women diagnosed with diabetes before pregnancy

Abstracts, letters, editorials and commentaries were also excluded.

### Search methods for identification of studies

The search strategy (MEDLINE version provided in the Additional file 1) was developed using a combination of Medical Subject Headings terms centred around three key areas: i) gestational diabetes mellitus ii) diagnostic testing for gestational diabetes mellitus and iii) patient experiences. The Systematic Review Accelerator software was used to translate the search strategy for each of the different databases and to remove duplicated articles [21]. We searched CINAHL, EMBASE, MEDLINE and PsycINFO databases from inception to April 2018. Forward and backward citation searching of included studies was conducted.

### Selection process

A single reviewer (LC) screened the titles and abstracts of retrieved references using Endnote Version X7.7.1. Potentially eligible full-texts were independently reviewed by LC and RS with conflicts resolved via discussion. Two full-text studies published in Portuguese were first translated using Google Translate and then validated by a researcher with both spoken and written Portuguese language skills located within our research network.

### Data extraction

All data labelled as results or findings including themes, categories, theories were extracted and imported into NVivo Version 12 by LC. Study characteristics were extracted by LC which included study location, reported research aims, study design, methodology and the analytical approach. Information about the diagnostic criteria used to determine GDM in women was also extracted.

### Data synthesis and analysis

To synthesise the findings, we used a thematic synthesis described by Thomas and Harden [22]. Thematic synthesis has the potential for conclusions to be drawn based on common aspects across otherwise heterogeneous studies and produce findings that directly inform health practitioners [22, 23]. Coding was inductive, with codes derived from the data. First, extracted text relevant to patient experiences and perspectives was coded line by line. A subset of studies (*n* = 5) were coded independently by LC and RS to develop a coding framework. Disagreements were resolved by discussion. LC and RS coded a further subset (*n* = 4) and established an inter-rater reliability of Kappa = 0.87. Following this, LC applied the coding framework to the remaining studies. New codes were iteratively developed as new concepts arose.

Second, relationships between the codes were identified by LC to form the basis of descriptive themes across the studies. Similar codes were grouped to generate themes and less frequently used codes were classified into sub-themes. In the final stage, analytical themes were developed to ‘go beyond’ the primary studies to amalgamate and interpret the findings. The relevant quotes to support each theme were tabulated.

### Quality assessment

As recommended by the Cochrane Qualitative Research Methods Group, we assessed the quality of the included studies using the Critical Appraisal Skills Programme Qualitative Checklist (CASP). This tool uses a systematic approach to appraise three key areas: study validity, an evaluation of methodological quality, and an assessment of external validity [24]. Critical appraisal was conducted by one reviewer (LC) for all studies, with second reviewer appraisal (RS) for a sub-set of included papers. The findings from the two reviewers were compared and any contrasting items were discussed and re-reviewed.

## Results

**Fig. 1:**
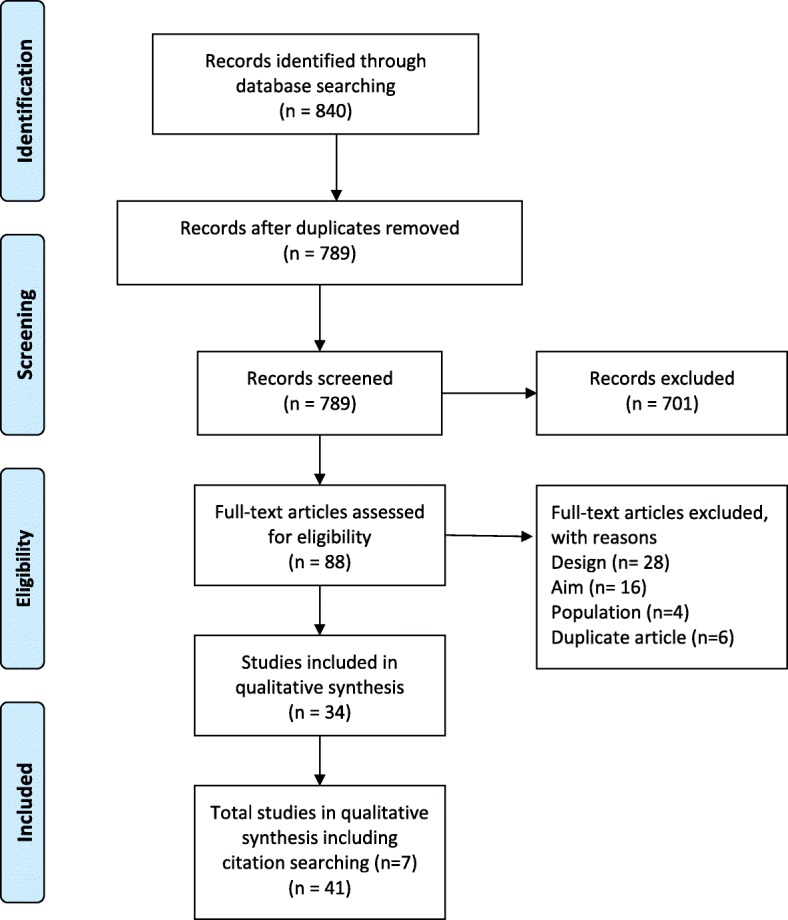
Prima flow diagram

The search identified 840 studies. After deduplication and screening of titles and abstracts 88 full-text articles were assessed (Fig. 1). Seven further articles were identified through citation searching. Data were extracted from 41 studies meeting eligibility criteria and were included in the review [11–13, 19, 25–61].

### Study characteristics

The studies reflected a variety of sampling methods and data collection methods. For example, interviews were conducted in 34 studies [10, 12, 13, 25, 27, 28, 30–32, 34–36, 38–50, 52–58, 60, 61], focus group methods were used in three [19, 32, 37], and interviews and focus groups were used in two studies [29, 51]. Two studies used a mixed method approach [26, 59]. The sample sizes ranged from 6 to 57 women. Eighteen studies were conducted in Europe, 10 in Australia, 9 in North America, and 2 studies each in Asia and South America. Table 1 details the characteristics of the included studies.

**Table 1:** Characteristics of included studies

Author/Date/country	Aim of study	Method of data collection/point of data collection	Conceptual basis underlying the study (e.g. thematic analysis, grounded theory)	Participants/Recruitment/N/Population description
Studies collecting data during pregnancy				
Carolan/2013 [29] Australia	To understand the experiences of women self-managing GDM	Phone interview, face-to-face interview and focus group	Thematic analysis	Pregnant women with a diagnosis of GDM/purposive sample/ *N* = 15 Caucasian, Asian, South Asian, Indian and Arabic
Carolan-Olah et al./2017 [12] Australia	To explore the experiences of a group of Hispanic women of Mexican origin who had been diagnosed with GDM	Semi-structured interviews	Thematic analysis	Pregnant Hispanic women with a diagnosis of GDM/convenience sample/ *N* = 18 Hispanic women of Mexican origin
Doran/ 2008 [30] Australia	To explore lifestyle changes during pregnancy and post-partum with women who had experienced a pregnancy complicated by GDM	Interviews	Thematic analysis	Pregnant women with GDM and women who has accessed centres for GDM management within the past 18 months/purposive sample/ *N* = 38 Pacific Islanders
Hjelm et al./2005 [41] Sweden	The aim of the present study was to compare beliefs about health and illness in women with GDM born in Swedish and in the Middle East	Semi-structured interviews	Thematic analysis	Pregnant women with GDM Interviews conducted at weeks 34–38/consecutive sample/ *N* = 27 (Sweden = 13) Swedish/Middle Eastern
Persson et al./2010 [13] Sweden	To describe pregnant women’s experiences of acquiring and living with GDM during pregnancy	Semi-structured interviews	Grounded theory	Pregnant women with GDM/convenience sample *N* = 10 Swedish
Kaptein et al./2015 [45] Canada	To gain insight into the reactions and experiences of women from multiple ethnic background diagnosed with GDM	Semi-structured telephone interviews	Thematic analysis	Pregnant women with a diagnosis of GDM/consecutive sample/ *N* = 19 Non-Caucasian (79%)
Trutnovsky et al./2012 [59] Austria	To explore concerns, treatment motivation, mood state, QoL, and treatment satisfaction of women treated for GDM.	Semi-structured interviews and survey	Thematic analysis	Pregnant women with GDM/convenience sample/ *N* = 45 Caucasian
Wah et al./2018 [60] Australia	To explore the understanding and self-management experiences of GDM among Chinese migrants	Semi-structured face-to-face interviews	Thematic analysis	Pregnant migrants of China ethnicity residing in Australia with a diagnosis of GDM/convenience sample/ *N* = 18 Chinese
Salomon et Soares/2004 [55] Portugal	To understand how gestational diabetes patients experience the impact of this diagnosis during pregnancy and of significance they attribute to the disease	Semi-structured interviews	Content analysis	Pregnant women with a diagnosis of GDM/unclear *N* = 9 Not reported
Hui et al./2014 [44] Canada	To explore the stress and anxiety experienced during dietary management for women with GDM	Food choice map semi structured interview *Interviews conducted at 26–28 weeks gestation*	Not specifically reported, described as thematic Themes	Pregnant women with diagnosis of GDM/purposive sample/ *N* = 30 Caucasian, Asian, African, and Aboriginal
Hjelm et al. 2012 [42] Sweden	Explore beliefs about health and illness in women with gestational diabetes living in Sweden and born in Sweden or Africa	Semi-structured interviews *Interviews conducted at weeks 34–38*	Categories with description extract	Pregnant women with a diagnosis of GDM/consecutive sample/ *N* = 23 (*N* = 13, Sweden) Swedish/African
Hjelm et al. 2008 [43] Sweden	To explore beliefs about health, illness and health care in women with gestational diabetes mellitus (GDM) managed in two different organisations based on diabetology or obstetrics	Semi-structured interviews *Interviews conducted at gestational weeks 34–38*	Thematic analysis	Women with a diagnosis of GDM/consecutive sample/ *N* = 23 Swedish/African
Hirst et al. 2012 [37] Vietnam	To determine attitudes and health behaviours of pregnant women with GDM in Vietnam	Focus groups	Thematic analysis	Women with a diagnosis of GDM/purposive sample (Women sampled at gestational ages 28–38 weeks) *N* = 34 Vietnamese
Han et al. 2015 [36] Australia	To explore women’s experiences after being diagnosed with borderline GDM	Semi-structured interviews	Content analysis Categories	Women with a diagnosis of borderline GDM Borderline GDM as a positive 50 g OGCT (1 h venous plasma glucose ≥7.8 mmol/L) followed by abnormal oral75g OGTT (fasting venous plasma glucose < 5.5 mmol/L and a 2 h glucose < 7.8 mmol/L) Eligible if they were participants in the IDEAL study/purposive sample/ *N* = 22 Caucasian and Asian
Ge, Wikby et al. 2016 [35] Sweden	To explore beliefs about illness and health and self-care behaviour among urban Chinese women	Semi-structured interviews	Content analysis Categories	Pregnant women with diagnosis of GDM, 34-38th gestational weeks/purposive sample/ *N* = 17 Chinese
Ge, Albin et al. 2016 [34] Sweden	To explore beliefs about health and illness and health-related behaviours among Chinese women with GDM in a Chinese sociocultural context.	Semi-structured interviews	Content analysis Categories	Pregnant women with a diagnosis of GDM, 34-38th gestational weeks/purposive sample/ *N* = 15 Chinese
Bandyopadhyay et al. 2011 [28] Australia	To explore the experiences and understanding of South Asian women after a diagnosis of GDM	Face-to-face interviews	Not specifically reported, described as thematic analysis Themes	South Asian women diagnosed with GDM/convenience sample/ *N* = 17 South Asian
Araujo et al./2013 [26] Brazil	To understand the significance of the experiences of women with gestational diabetes mellitus	Open interviews and participant drawings *4 women in 1st trimester, 3 in 2nd trimester & 3 in 3rd trimester*	Not specifically reported, described as thematic analysis	Women with GDM diagnosis/convenience sample/ *N* = 12 South American
Evan et Brien 2005 [12] Canada	To gain an in-depth understanding of GDM as experienced by pregnant women	Interviews *Interviews conducted prior to delivery and 6–8 weeks postpartum*	Thematic analysis	Women with GDM diagnosis/purposive sample/ *N* = 12 Caucasian
Studies collecting data within the 1st 12 months post-natal				
Bandyopadhyay et al./2015 [27] Australia	To capture in-depth exploration of the experiences and perspectives on postpartum glucose tolerance test screening of South Asian women diagnosed with GDM	Interviews *Interviews were conducted antenatally after diagnosis, after birth, 9 weeks to 52 weeks*	Thematic analysis	South Asian women with diagnosis of GDM/convenience sample/ *N* = 40 South Asian
Draffin et al./2016 [19] United Kingdom	To explore the concerns, needs and knowledge of women diagnosed with GDM	Focus groups	Thematic analysis	Pregnant women with a diagnosis of GDM or a history of GDM within past 12 months/convenience sample/ *N* = 19 White, Black African, Pakistani Latin American, Bangladeshi, Indian
Doran et Davis 2010 [31] Tongan	To explore GDM in Tonga, with women who experienced GDM and health professionals who worked in the GDM/diabetes area	Semi-structured face to face interviews	Not specifically reported, described as thematic analysis Themes	Women who had experienced GDM in the previous 12 months /unclear/ *N* = 11 Pacific Islanders
Figueroa Gray et al./2017 [33] USA	To foreground women’s experience with insulin and oral hypoglycemic agents.	Focus group	Thematic analysis	Women with GDM history and completed at least one prescription for insulin or oral hypoglycaemic medication during pregnancy within past 3 years/purposive sample/ *N* = 16 Caucasian, African American, Asian, Hispanic or Latina
Hjelm et al. 2009 [40] Sweden	To explore beliefs about health and illness 3 month postpartum in women born in Sweden and the Middle East, and to study whether they perceive gestational diabetes mellitus as a prediabetic condition	Interviews	Headings and descriptions Divided into Middle-Eastern born and Swedish born women	Women 3 months postpartum who had previously had GDM /consecutive sample/ *N* = 27 Swedish and Middle Eastern
Hjelm et al./2012 [42] Sweden	To explore the development over time of belief about health, illness and health care in migrant women with gestational diabetes born in the Middle East and living in Sweden	Semi-structured interviews *Interviews conducted at weeks 34–38, three and 14 months after delivery*	Content analysis	Middle Eastern women with a diagnosis of GDM/consecutive sample/ *N* = 14 Swedish and Middle Eastern
Hjelm et al. 2018 [39] Sweden	To explore the development over time, during and after pregnancy, of beliefs about health, illness and healthcare in migrant women with GDM born in Africa living in Sweden	Semi-structured *Interviews conducted in gestational weeks 34–38 and 3 and 14 months after delivery*	Framework analysis using the Health Belief Model	Women with a diagnosis of GDM/convenience sample/ *N* = 9 African
Kilgour et al./2015 [46] Australia	To explore and assess women’s communication experiences of postnatal GDM follow-up	Interviews *Interviews at 12–16 weeks after birth*	Thematic analysis	Women with GDM diagnosis/“theoretical sample”/ *N* = 13 Caucasian, Asian and Indian
Lawson et Rajaram/1994 [47] USA	To explore the meaning women attach to GDM	Semi-structured interviews *Interviews once prenatally and again at 6 weeks*	Thematic analysis	Women with diagnosis of GDM/purposive sample/ *N* = 17 Caucasian, Black and Asian-American
Neufeld/2011 [49] Canada	To describe how aboriginal women in an urban setting perceive dietary treatment recommendations associated with GDM	Interviews	Thematic analysis	Aboriginal women with GDM or a previous diagnosis of GDM within past 5 years/convenience sample/ *N* = 29 Aboriginal
Svensson et al./2018 [56] Denmark	To examine how Danish women with a history of GDM experience the transition from a GDM-affected pregnancy to the postpartum period	Interviews *Interviews within 3–5 months after delivery*	Content analysis Themes	Women diagnosed with GDM/convenience sample/ *N* = 6 Caucasian
Tang et al./2015 [57] USA	To gain insight of Hispanic and African-American women’s views on prevention of T2DM after GDM.	Semi-structured interviews	Thematic analysis	Women with a history of GDM (within 12 months of delivery at the time of initial contact)/purposive sample/ *N* = 23 African-American
Whitty-Rogers et al./2016 [61] Canada	To explore Mi’kmaq women’s experiences with GDM.	Conversational interviews	Hermeneutic phenomenology Themes	Mi’kmaq women with history of GDM/purposive and snowballing sample/ *N* = 9 Aboriginal
Studies collecting data at follow up screening for Type II diabetes				
Abraham et Wilk/2014 [25] USA	To explore the lived experiences of women in rural communities with GDM and potentially gain insight into the low reported return rates for PPG testing	Semi-structured interviews	Phenomenological approach Themes	Women with a history of GDM in the last 2 to 5 years/purposive and snowballing sample/ *N* = 10 Caucasian
Eades et al./2018 [32] UK	To explore experiences, knowledge and perceptions of women with GDM to inform the design of interventions to prevent or delay Type 2 diabetes	Semi-structured interviews	Theoretical framework – Self-Regulation Themes	Women with history of GDM diagnosis, within 1-year post delivery/convenience sample/ *N* = 16 Caucasian, Asian, Black and African
Nielsen et al./2015 [50] Denmark	To improve our understanding of how women with gestational diabetes experience the treatment and care offered by a regional health service. To understand how the women’s experiences influenced their subsequent participation in follow-up screening.	Semi-structured interviews	Thematic analysis	Women with a previous diagnosis of GDM within 1–2 years after birth/convenience sample/ *N* = 7 Caucasian and Asian
Parsons et al./2018 [51] UK	To describe the experiences of women from a demographically diverse population of their GDM and GDM care, to help inform healthcare delivery for women both during and after pregnancy	Interviews and focus groups	Framework analysisThemes	Women with a previous diagnosis of GDM (within past 5 years)/purposive sample/ *N* = 50 Black, Caucasian, and Asian
Razee et al./2010 [54] Australia	To explore the beliefs, attitudes, social support, environmental influences and other factors related to diabetes risk behaviours among Arabic, Cantonese/Mandarin, and English speaking women with recent GDM	Semi-structured interviews	Not specifically reported, described as thematic analysis	Women who had completed a GDM pregnancy in the previous 6–36 months/purposive sample/ *N* = 57 Middle Eastern, Chinese and White Australian
Rafii et al./2017 [53] Iran	To understand Iranian women’s experiences in diabetes screening after childbirth	Semi-structured interviews *Interviews at 6–21 months postpartum*	Grounded theory methodology Themes and sub-themes	Women with previous GDM diagnosis /purposive sample/ *N* = 22 Asian
Tierney et al./2015 [58] Ireland	To assess the lifestyle behaviours undertaken by a group of women both during and after their GDM pregnancy	Semi-structured interview	Thematic analysis	Women with a history of GDM in the previous 3.6–6.6 years/convenience sample/ *N* = 13 Not reported
Pennington et al./2017 [52] Australia	To explore the views of GPs and women who have had GDM	Semi-structured interviews	Content analysis	Women with a history of GDM/purposive sample/ Timeframe not reported N = 16 Not reported
Lie et al./2013 [48] United Kingdom	To explore factors influencing post-natal health behaviours following the experience of gestational diabetes	Semi-structured interviews	Framework analysis	Women with a history of GDM within the last 2 years/purposive sample/ *N* = 37 Caucasian and non- Caucasian

### Quality appraisal

Most studies were assessed as high quality (Additional file 1: Table S2). Study aims were stated in all but one study [47]. As the purpose of all included studies was to explore or gain knowledge, opinions or attitudes about GDM, the qualitative methods employed in all the studies were appropriate. Different study designs were used and in some cases the lack of reporting details made judgments of the appropriateness of study methods difficult. Data were collected in a way that addressed the research issue, however, a few authors did not discuss or report details such as saturation of data [31, 47, 56, 59]. The relationship between researcher and participants was considered in only two studies [51, 61]. Appropriateness of data analysis was assessed as “unclear” when there was a lack of details about how themes were derived.

### Thematic analyses

Eight themes were generated from the data synthesis: 1. initial psychological impact; 2. communicating the diagnosis; 3. knowledge of GDM; 4. risk perception; 5. management of GDM; 6. burden of GDM; 7. social support; and 8. gaining control. The relevant quotes to support each theme are presented in Table 2.

**Table 2:** Data to support identified themes

No	Theme	Supporting data
1	Initial psychological impact	*‘I was very surprised and very upset to be diagnosed. I felt a little bit of a failure.’* [31] *‘You actually feel guilty, right? Because this baby hasn’t asked for this; and what if the baby comes out and has some kind of disease? Then it’s my fault.*’ [58] *‘GDM was a hidden blessing for me... GDM can go away after you have the baby but diabetes is not so easily fixable …I am much more aware of* [the] *need to prevent it.’* [32] *‘It’s also good with my diabetes diet I ended up weighing pretty much the same at the end of the pregnancy as I did at the beginning.’* [31]
2	Communicating the diagnosis	*‘Like they don’t have time to sit there and talk to you about what to do about it [GDM], but they are always in a hurry [………] so I just don’t bring it up and they don’t bring it up; so you just get checked out and leave.’* [26] *‘They sent me off to the dietician and I came out depressed....I went “nuh - if I eat what you’re telling me”... and she was telling me I had to eat carbs with every meal. I knew it wouldn’t control my BSL [blood sugar level]...hadn’t they read the latest research about a high protein diet being more beneficial that a high carb diet?* [32]. *‘scared that if the sugar was too high they would take the baby out* [33]. *‘Nobody told me anything about type 2 diabetes...they were so focused on the immediate pregnancy problems; within a medical model...OK, if we don’t get it under control, we will just put you on insulin... just a drug solution.’* [32] ‘*They [hospital dieticians] don’t have much information on for example some Chinese dishes, so I would go on the Internet and check if they’d raise my blood sugar*. *.*. *but even on the Internet, it’s hard to find information like that*.’ [62]
3	Knowledge of GDM	*‘I thought it was the end of the world because I didn’t fully understand it’* [27]. *‘At first I thought: it must be because I ate a lot sweet, overweight ... but then I was talking to the girl, she said it must be family, right*?*?’* [57] *‘My husband, he always knew me like this ... is ... healthy, without any illness. [...] Because he is kind of ignorant in these matters, you know. He thinks that diabetes ... that I’m dying! ... And my boy too, is half pensive, cautious, thinking I’m going to die.’* [57] *‘I did not know that rice can have that much sugar. That’s one thing really, really surprised me. I’m thinking that sugar normally comes from cakes and chocolate.*’ [31] *I’ve eaten something and I’ve thought my reading’s not going to be good after this and it’s been fine. So it was mainly getting my head around it’s not just the sugars. Like Special K for example. That was on my list of things that I could have and then the lady (educator) said, ‘What did you have for breakfast? And I said I had Special K. She said, ‘it obviously doesn’t work for you. So you either have to up your insulin, if you want to just keep eating Special K, or just stop eating it.’* [31] *‘You’d think, okay, well this will be good; this will be fine for me to eat. Then I will check my sugars 2 h later and it would not. I would be why? That’s not okay. It was disappointing, and it was definitely stressful, like it was just really not fun.’* [46] *If the mother breastfeeds her baby the ‘diabetes factor’ may transmit to the baby and it’s no good. It may make the baby have the same disease afterwards’* [39] *‘I’m concerned about that (transmission of diabetes to the infant) of course’* [39]
4	Risk perception	*‘coming back as borderline gestational diabetes wasn’t such an issue as having full-blown diabetes...and I don’t worry about it.’* [38] *‘I’m afraid this diet won’t provide enough nutrients for the baby, but the doctor told me to do that’* [38] *‘since, after giving birth and everything’s back to normal so I’ve sort of been making up for lost time a little bit with all the chocolate I couldn’t have.*’ [52] *‘It’s actually quite odd that during birth, they monitored everything closely, and as soon as I had delivered they served me a piece of white bread.*’ [52]
5	Managing GDM	*‘It is frustrating still when you watch your carbs, you portion it and your reading is still high, almost every day.’* [46] *‘I’ve been doing everything right. My sugar is so unstable. The highest reading was 202 with my insulin. I have eaten the right foods, exercised, and tested my glucose levels four times a day. I had some high ones of 151. When my insulin dosages are increased, I am more depressed. I feel worthless.*’ [49] *‘I really wanted to control it through my diet and exercise. I was strongly motivated to do all I could so I didn’t have to introduce another needle.... But when I went on insulin I relaxed.*’ [32] *‘..my baby might die if I’m not on [a] diet.*’ [39] *‘Well, you are deviant from others. It’s like a functional handicap in that aspect’* [15]. *‘I’m a full-time working mom, so I had to carry a little refrigerator with me with the insulin to work every day and on weekends to the restaurant and have to hide in the bathroom. It made me feel like I’m totally an illegal person.’* [35]
6	Burden of GDM	*‘The whole pressure with the whole everything, it really did affect me and I think it’s probably one of the worst times I’ve had in my life actually.*’ [53] *‘I would have crashes where I’d be driving on the freeway, and I’m at a crazy low number, and I have to try to find some candy or something in my car. So it was very frustrating.*’ [35] *‘because it is really ugly to have, in fact I wanted to have another baby and since I got this I do not want to anymore.’* [14]
7	Social support	*‘Well, I wouldn’t have done it without my partner like because he was like, “Up, eat now, insulin,” you know, and I would be, “Yeah, I’m going to get up in 20 min and I’m going to do this,” and he was like, “Now, eat, your insulin,” you know.’* [21] *‘he’s now cooking more for me and he’s healthier because he doesn’t, because if he’s going to eat junk food that’s just going to make me jealous. So he’s kind of trying to eat healthy as well for me.*’ [31] *‘My mother-in-law phoned relatives and told the villagers that my baby was not healthy because I had GDM...’* [37]
8	Gaining control	*‘You have an active role and you can take charge of what’s going on rather than just roll along.’* [12] *‘I believe in not giving in to diabetes. I will take care of myself and control the diabetes.*’ [49] *‘I looked at herbal remedies because that’s something that [laughing], you know, you think is quite safe* [21]

#### Initial psychological impact

When initially diagnosed with GDM, most women reported reactions such as self-blame, failure, fear, sadness, concern and confusion. Women often focused on the uncertainty of diagnostic prognosis and some considered it to be a life-altering experience. Some women felt lost and unsure what to do next. Often women felt an overwhelming sense of vulnerability and guilt. In some cases, the diagnosis was received positively and was viewed as an opportunity for lifestyle improvements. For example, some women viewed the diagnosis as a ‘*wake up’* call and were grateful for the chance to intervene and potentially prevent adverse outcomes for their babies and themselves. Some women viewed gaining less weight than expected during their pregnancy as a benefit of having a GDM diagnosis.

#### Communicating the diagnosis

Communication with healthcare professionals (HCPs) and their families was a common theme throughout the findings of the included studies. Generally, the level and quality of communication with HCPs was mixed – with some women reporting positive and informative encounters, while others described brief encounters with overly technical language and unsupportive consultations. The main issues were limited time available to spend with the HCP, lack of continuity of care and lack of understanding about the role of the HCP at follow-up. In some instances, women felt that GDM was not a topic that HCPs were keen to discuss -*‘the nurses, they never talked to me about my gestational diabetes’.* [23] The level and quality of information provided was often conflicting, confusing or insufficient. Areas of contention were appropriate foods and the dietary changes that should be made.

Some women formed a dependency on HCPs to know what to do and on the electronic reminders for follow-up appointments and monitoring. Often women reported having no choice in treatment resulting in them feeling threatened and frustrated. Often women were automatically booked in for a caesarean section without consultation or lived in fear of this occurring. One woman referred to GDM as being over medicalised. Receiving limited information also prompted women to independently seek information about the impact and management of GDM from other sources such as the internet. However, some women found the internet limited for specific information or confusing.

#### Knowledge of GDM

Women had varying levels of understanding of GDM which impacted on their initial reaction to the diagnosis. Those who were able to explain the cause of GDM were able to process and accept the diagnosis more readily than those who had little understanding of GDM, or were confused as to how GDM occurred. Lack of knowledge also extended to and impacted on relatives. Some women stated that they would have preferred to be more prepared to receive the diagnosis by having early knowledge about the testing for diabetes. Women reported being on a steep learning curve, especially the onerous approach of dietary trial and error whereby women learnt what foods would increase their blood glucose level (BGL) and what food to avoid. Women also reported challenges in adopting new habits to manage their GDM, including understanding food labels and nutritional values of food. Often this required a trial and error approach. There was also a lack of understanding about the impact of GDM on their baby with some women believing it would be transmitted to their baby via breastmilk.

#### Risk perception

Women’s perception of risk were reported before the diagnosis of GDM, after they were diagnosed in pregnancy, and after the delivery. Some women attempted to understand their level of risk in context of family history. Some were very surprised by the diagnosis, especially if they were asymptomatic; and some women found it difficult to come to terms with the diagnosis. There was uncertainty about the severity of the condition. Some women considered the condition to be mild, downplaying the disease and believing that too much ‘*fuss’* was being made about GDM and other women doubted the diagnosis and its seriousness. Women often discussed: the adverse effects that GDM would have on her baby; frustration that the focus was on risks to the baby and less so them; their worry about the consequences for the future; and questioned the impact of insulin on the baby. Women worried that their diet was too restrictive for their growing baby and would not provide the nutrients that the baby required. Some women held the view that GDM was a temporary condition and would disappear once the baby was born, and many women reverted to old eating habits after the baby’s birth. Often women referred to the birth as a ‘*moment of truth*’ or as an endpoint to their GDM. This was also reflected in the level of care that the women received after the birth of their baby.

#### Managing GDM

Dietary management-related stress was commonly reported amongst interviewed women and was experienced by both insulin and non-insulin users. Stress and frustrations often occurred as a consequence of an unexpected abnormal blood glucose reading following strict adherence to dietary advice. Maintaining stable BGL was an ongoing struggle and in some cases the burden proved too much, with a few women ceasing employment. Insulin users described the process as a ‘*roller coaster*’ as well as the emotional and physical discomfort of injecting, while non-insulin users often became obsessed with a well-controlled diet, with some women viewing this as a way to avoid the use of insulin. Conversely, some women felt relieved when they were transitioned onto insulin, as it reduced the need for dietary restriction.

Making lifestyle changes was considered stringent and restrictive by the majority of women, and for some required ‘*major restructuring’* to their diet and daily routines to incorporate exercise. Some women reported extreme behaviours, including falsifying blood glucose readings, self-starvation and hiding their condition, including from family members. Often the impact of non-adherence to lifestyle changes resulted in guilt and belief that the baby would know they have cheated. Other pregnancy related ailments and the need to care for other children interfered with the ability to make the required changes. Women who had a specific culture-related diet discussed the impact and difficulty of applying or tailoring the dietary recommendations to their diet.

The key motivator to making required lifestyle changes, despite the associated hardships, was to minimise the risks to their unborn baby. Women prioritised the health of the baby over their own health and were willing to do anything to ensure that the health of their baby was not compromised. Over time, management of the GDM became a part of their normal routine for many women. However, some women expressed a desire to have a ‘*normal’* pregnancy similar to their friends, discussing that a diagnosis of GDM made them feel as though their pregnancy was atypical, leading to defining their pregnancy as ‘*abnormal*’ or as an ‘*illness*’. For one woman, it made her feel like an ‘*illegal’* person.

#### Burden of GDM

Women reported that a diagnosis of GDM came with extra responsibility, which added pressure whilst trying to juggle life commitments such as work, childcare, and daily living responsibilities. Monitoring and treating GDM placed burden on women’s daily routines and most woman agreed that taking BGL measurements was time consuming and disruptive. There was a constant need to prudently plan meals and co-ordinate the attendance at additional hospital appointments, all of which were considered time intensive, especially with travel and wait times. Women expressed that GDM consumed a lot of their thinking time e.g., ‘*I think about diabetes everyday’* and felt that they had to acknowledge GDM all the time and became ‘*super-conscious’*. In some instances, women reported a GDM diagnosis took away some of the ‘*joy of pregnancy*’*.* One woman described her pregnancy as a ‘*misfortune’*. Women mentioned the financial burden of buying healthier food – *‘it would take lots of money just because it is so expensive to eat healthy’.* [25] Women also considered the physical burden of GDM such as fatigue and the side effects of treatment such as insulin. There was a longer-term impact on family planning, where in some cases women decided not to have another child because they were fearful of enduring a similar restrictive and stressful pregnancy due to GDM.

#### Social support

Social support, including family and HCP support, was an important aspect for women during their experience of a GDM diagnosis. Changes in lifestyle often had an overflow effect, with other family members adopting healthier lifestyles. Women not in their country of birth, and without family, often reported feeling isolated and lonely. Disappointment and isolation were also expressed by some women when they perceived a lack of healthcare system support. This often occurred postnatally when the expectations of postpartum care were high, however, in reality, support was absent. In some cases, women were stigmatised by their families and in a few cases received undesirable feedback that they were not doing enough to protect their unborn child.

#### Gaining control

Control was a frequently used word when women described living with and managing a GDM diagnosis. Initially women reported a lack of control especially over their emotions, however, over time women transitioned from feeling like a victim of diabetes, to being active agents in controlling their GDM. The terms ‘*balance’* and ‘*adjustment’* were used to describe how some women tried to offset the strict compliance and active self-management with reducing their risk to their unborn baby and their own future risk of developing diabetes after pregnancy. Some women reported feeling empowered as their pregnancies progressed, especially when they gained more knowledge about GDM and what action they could take to accept and make sense of the diagnosis. Taking control included realising the changes that were required to their lifestyle, self-initiated care, and self-education. Often investigating alternative options, such as natural remedies outside those recommended by HCPs, provided women with some autonomy in managing their condition and some believed that it was a safer option to medication.

## Discussion

### Summary of main findings

This synthesis of the qualitative evidence of women’s experiences of being diagnosed with GDM highlighted the psychosocial consequences a diagnosis of GDM can have on women. The purported benefits of a GDM diagnosis identified from our review, were largely behavioural and included an opportunity to improve health, prevent excessive weight gain, control weight during pregnancy, and prompts to make healthy eating changes. However, the purported harms included the added responsibility (eating regimens, appointments), financial constraints (expensive food, medical bills), and conflicts with their cultural practices (alternative eating, lack of information about traditional food). The psychosocial consequences were wide reaching and often resulted in significant social isolation with women only sharing their diagnosis with partners. Furthermore, there were a few reports of over-medicalisation due to a GDM diagnosis, with the perception that HCPs were often authoritarian, focusing on physiological aspects, with little attempt to involve women in decision making. This is noteworthy considering a non-GDM pregnancy has already come under scrutiny as being over-medicalised with increasing levels of unnecessary intervention [62].

Women from studies included in our review frequently reported inconsistent information provision. Limited GDM information provision has been identified in another systematic review regarding healthcare seeking for GDM during the postpartum period [63]. In contrast, findings from another study which aimed to evaluate satisfaction with obtaining a diagnosis of GDM concluded that the majority of women were satisfied with their experience of being diagnosed [64]. Further, women in the latter study associated poor GDM control with perinatal complications and an increased risk of type 2 diabetes following pregnancy [64].

Another key finding from this review was low awareness of the potential risks of GDM, particularly in the long-term. Low health literacy levels could be one factor to explain knowledge deficits and understanding of GDM, especially given the sociodemographic diverse population included in this review. One study found that low literacy among disadvantaged women had a significant impact on their understanding of GDM information [65]. Other research found that women who live in an English-speaking country but primarily speak a non-English language, have lower rates of dietary awareness compared with their English speaking counterparts, and this may affect compliance to dietary interventions [66]. Therefore, it is important that new educational interventions are developed to target those with lower health literacy as well as cultural factors when diagnosing and managing multi-ethnic populations with GDM [66].

Interestingly, women with a borderline diagnosis of GDM did not seem as concerned as other women and in some cases were dismissive of the diagnosis and the potential consequences. Similarly, in a study which specifically included women with a borderline diagnosis of GDM, the majority of women reported that they were not worried by the diagnosis [67]. For some women, the potential transitory nature of GDM was emphasised and some reported that it didn’t seem like a real illness. The diagnostic criteria for GDM has previously been compared with the established criteria used to classify a condition as a disease. This comparison revealed disparity which Goer, in 1996, used to suggest that GDM did not pose a serious health risk, was neither easily nor accurately diagnosed, was not treated effectively and that treatment outweighed the risks of the condition [68]. Therefore, the levels of heightened psychological distress as reported by the women in our review, may actually be unnecessary and others have gone as far as saying that GDM is an example of ‘obstetric iatrogenesis’ [69].

The findings of this review did underline a few unmet service needs with recurring themes around the lack of individualised care and its continuity, lack of choice regarding important aspects of care such as birthing options, and the scarcity of comprehensive follow-up. There was a sense of abandonment amongst women after delivery in that they had experienced intensive intervention and then nothing. This could be viewed as a missed opportunity to capitalise on the motivation to make changes during pregnancy. Researchers have previously highlighted that adherence to postpartum screening and continued lifestyle modifications to prevent future diabetes seems to dissipate after birth, possibly because the driver to protect their unborn child is no longer there [70].

The studies included in our review had participants of varying cultures sampled from countries with different GDM definitions. However, there appeared no difference in the qualitative outcomes between studies/countries. In our review, the experiences of women diagnosed with GDM suggest psychosocial harms appear to outweigh the qualitative benefits. Quantitative studies [14, 15] that report prevalence increases in GDM after the IADSPG [71] definition changed, also report minimal improvements to maternal and infant physical outcomes.

This synthesis of women’s experiences of a GDM diagnosis could be used to inform the content of communication materials both before and after a GDM diagnosis. For example, an awareness of GDM testing and basic information including cultural adaptations to dietary guidelines and addressing misconceptions around breastfeeding. There is also an opportunity for HCPs to use teachable moments with women who have been identified at risk of developing type 2 diabetes post-pregnancy and offer supportive, effective advice about lifestyle changes. This is particularly relevant considering a previous review highlighted a significant time is spent in sedentary behaviour during pregnancy [72]. A study which examined HCPs views of healthcare provision to women with GDM showed that HCPs themselves perceived that there was a shortfall in GDM education [73]. There are also signals for service improvement and potential for service redesign, such as increasing community-delivered care for women diagnosed with GDM. This would assist in alleviating the burden on women to attend hospital appointments and potentially offer flexible appointment times. Follow-up appointments post-pregnancy could be made with consideration of other appointments such as maternal and child health milestones and breastfeeding weaning classes, and could also focused on healthy eating for both mother and baby.

### Strengths and limitations

This systematic review included studies with women of different demographic characteristics and multicultural samples. The themes identified were represented in the majority of studies which increased the internal validity. The relatively high participation rate in the included studies, and that most studies were conducted during pregnancy or shortly after delivery, contributes to the external validity of our study. Although some participants were interviewed antenatally and some postnatally, this distribution over different gestational stages assists the generalisability of the study findings.

The comparison of coding between authors, discussion of the results and reaching consensus was a robust approach to improve the credibility of the results. Overall, the quality of most studies was good, however, a third of the studies used convenience methods to recruit participants which could contribute to sampling bias and limit the external validity of our findings. Only two studies adequately described the facilitator’s prior experience and the relationship between the participants and the facilitator/researcher. Unfortunately, this review did not capture the perception of HCPs which might be used to explain some of the behaviours and attitudes of the women, particularly in relation to communication of the diagnosis and information provision. Finally, although the data were collected from diverse populations, the majority of the countries in which research were conducted in were high-income countries, which could be considered to have more established and evidence-based healthcare systems than low-income countries.

### Further research

A previous study has suggested the need for more research on the benefits and harms of alternative treatment choices for women with GDM [33]. The findings from this review suggest a need for more investigation around the psychosocial benefits and harms of a diagnosis of GDM. Given some women viewed treatment of ‘borderline GDM’ as unimportant, a new model of care based on stratification or individual level of risk for pregnancy and birth complications could be further explored. This may reduce the need for all women to be labelled as having GDM and negate unnecessary anxiety and burden for those at the lower ‘borderline’ threshold. This would then potentially offer tailored treatment options, improve shared-decision making, and improve women’s knowledge about how a diagnosis of GDM might affect them.

## Conclusion

Consequences of a GDM diagnosis are multidimensional and highly contextual. Despite the psychosocial challenges frequently experienced, many women (driven by the innate response to safeguard their unborn baby) were able to gradually adapt to the required lifestyle changes and monitoring regimens. Perhaps a question is whether some of them should have to. There is opportunity to improve lifestyle and to assist the prevention of diabetes after pregnancy, however, this needs to be managed alongside the potential harms of a GDM diagnosis such as the negative psychological impact and social isolation. In the context of rising prevalence [14–17], potential minimal clinical [14–16] improvements, and the wide range of psychosocial experiences identified in this study, the findings of this review highlight the need for HCPs to consider the implications that a GDM diagnosis may have on women. It is essential that women diagnosed with GDM receive consistent evidence-based information and ongoing psychological and social support.

## Supplementary information

**Additional file 1: Table S1.** Enhancing Transparency in Reporting the Synthesis of Qualitative Research Guidelines Checklist. **Table S2.** Assessment of quality of included studies using the CASP tool.

## Data Availability

The datasets generated during the current systematic review are available from the lead author upon request.

## Abbreviations

BGL: Blood glucose level
CASP: Critical Appraisal Skills Programme Checklist (Qualitative)
ENTREQ: Enhancing Transparency in Reporting the Synthesis of Qualitative Research
GDM: Gestational diabetes mellitus
HAPO: Hyperglycemia and Adverse Pregnancy Outcomes
HCP: Health care professional
IADSPG: International Association of the Diabetes and Pregnancy Study Groups
