# Supplementary material for: Women’s experiences of a diagnosis of gestational diabetes mellitus: a systematic review
Source: BMC Pregnancy Childbirth. 2020 Feb 7;20:76. doi: 10.1186/s12884-020-2745-1 (PMC7006162; doi:10.1186/s12884-020-2745-1)
Supplement: Supplementary file 1 — Additional file 1: Table S1. Enhancing Transparency in Reporting the Synthesis of Qualitative Research Guidelines Checklist. Table S2. Assessment of quality of included studies using the CASP tool. [file 12884_2020_2745_MOESM1_ESM.docx]

**MEDLINE SEARCH STRATEGY**

exp Diabetes, Gestational/) OR GDM.tw. OR (gestation$ adj2 (diabet$ or DM or glucose intoleran$ or insulin resistan$)).mp. OR (pregnan$ adj3 (diabet$ or DM or glucose intoleran$ or insulin resistan$)).mp.) OR (maternal adj2 (diabet$ or DM or glyc?emia or hyperglyc? emia)).tw. OR (women adj2 (diabet* or DM or gly?emia or hyperglyc?emia)).tw. OR (Pregnancy in Diabetics/eh [Ethnology]) OR Pregnancy in Diabetics/px [Psychology] OR (hyperglyc?emia adj2 pregnan$).tw.

AND

OR mass screening/ OR prenatal diagnosis/ OR screen$.tw. OR ((prenatal or early) adj2 diagnosis).tw. OR Glucose Tolerance Test/ OR Glucose Intolerance/ OR Blood Glucose/ OR (glucose adj (tolerance or intolerance or challenge)).tw. OR OGTT.tw. OR GCT.tw. OR (fasting adj2 glucose).tw. OR Diabetes, Gestational/di [Diagnosis] OR Diabetes, Gestational/px [Psychology] OR Diabetes Mellitus, Type 2/di [Diagnosis] OR Diabetes Mellitus, Type 2/ep [Epidemiology]

AND

Patient Satisfaction/ OR patient$ experience$.mp. OR women$ experience$.mp. OR Attitude to Health/ OR Mothers/px [Psychology] OR Patient$ Perspective$.mp. OR (stress$ or uncertaint$ or anxiet$ or fear$ or distress$ or coping or well-being or anger or depress$).mp. OR Stress, Psychological/ OR Anxiety/co [Complications] OR Anxiety/px [Psychology] OR Beliefs.mp. OR Self-Management/ OR Self Care.mp. OR Health Knowledge, Attitudes, Practice.mp. OR experience$.mp. OR Life Style/ OR psychosocial issues.mp. OR psychosocial support systems/ OR Health Behavior/

AND

Pregnancy/ OR Prenatal Care/ or Maternal Health Services/ or HOSPITALS, MATERNITY/ or Infant, Newborn/ OR Postpartum Period/ or Postnatal Care/ OR WOMEN/ or PREGNANT WOMEN/ or Female.tw. OR 1 or 2 or 3 or 4 or 5 or 6 or 7 or 8 or 9

AND

Qualitative Research/ OR Interview/ OR (theme$ or thematic).mp. OR qualitative.af. OR Nursing Methodology Research/ OR questionnaire$.mp. OR ethnological research.mp. OR ethnograph$.mp. OR ethnonursing.af. OR phenomenol$.af. OR (grounded adj (theor$ or study or studies or research or analys?s)).af. OR (life stor$ or women* stor$).mp. OR (emic or etic or hermeneutic$ or heuristic$ or semiotic$).af. or (data adj1 saturat$).tw. or participant observ$.tw. OR (social construct$ or (postmodern$ or post-structural$) OR (post structural$ or poststructural$) OR post modern$ or post-modern$ or feminis$ or interpret$).mp. OR (humanistic or existential or experiential or paradigm$).mp. OR (field adj (study or studies or research)).tw. OR human science.tw. OR biographical method.tw. OR theoretical sampl$.af. OR ((purpos$ adj4 sampl$) or (focus adj group$)).af. OR (account or accounts or unstructured or openended or open ended or text$ or narrative$).mp. OR (life world or life-world or conversation analys?s or personal experience$ or theoretical saturation).mp. OR ((lived or life) adj experience$).mp. OR cluster sampl$.mp. OR observational method$.af. OR content analysis.af. OR (constant adj (comparative or comparison)).af. OR ((discourse$ or discurs$) adj3 analys?s).tw. OR narrative analys?s.af.

Table S1. Enhancing Transparency in Reporting the Synthesis of Qualitative Research Guidelines Checklist

| **No** | **Item** | **Guide and description** | **Page number** |
| --- | --- | --- | --- |
| 1 | Aim | State the research question the synthesis addresses. | 4 |
| 2 | Synthesis methodology | Identify the synthesis methodology or theoretical framework which underpins the synthesis, and describe the rationale for choice of methodology (e.g. meta-ethnography, thematic synthesis, critical interpretive synthesis, grounded theory synthesis, realist synthesis, meta-aggregation, meta-study, framework synthesis | 5-6 |
| 3 | Approach to searching | Indicate whether the search was pre-planned (comprehensive search strategies to seek all available studies) or iterative (to seek all available concepts until they theoretical saturation is achieved). | 5 |
| 4 | Inclusion criteria | Specify the inclusion/exclusion criteria (e.g. in terms of population, language, year limits, type of publication, study type). | 4-5 |
| 5 | Data sources | Describe the information sources used (e.g. electronic databases (MEDLINE, EMBASE, CINAHL, psycINFO, Econlit), grey literature databases (digital thesis, policy reports), relevant organisational websites, experts, information specialists, generic web searches (Google Scholar) hand searching, reference lists) and when the searches conducted; provide the rationale for using the data sources. | 5 |
| 6 | Electronic search strategy | Describe the literature search (e.g. provide electronic search strategies with population terms, clinical or health topic terms, experiential or social phenomena related terms, filters for qualitative research, and search limits). | 5 |
| 7 | Study screening methods | Describe the process of study screening and sifting (e.g. title, abstract and full text review, number of independent reviewers who screened studies). | 5 |
| 8 | Study characteristics | Present the characteristics of the included studies (e.g. year of publication, country, population, number of participants, data collection, methodology, analysis, research questions). | Table 1 |
| 9 | Study selection results | Identify the number of studies screened and provide reasons for study exclusion (e,g, for comprehensive searching, provide numbers of studies screened and reasons for exclusion indicated in a figure/flowchart; for iterative searching describe reasons for study exclusion and inclusion based on modifications t the research question and/or contribution to theory development). | 6 |
| 10 | Rationale for appraisal | Describe the rationale and approach used to appraise the included studies or selected findings (e.g. assessment of conduct (validity and robustness), assessment of reporting (transparency), assessment of content and utility of the findings). | 6 |
| 11 | Appraisal items | State the tools, frameworks and criteria used to appraise the studies or selected findings (e.g. Existing tools: CASP, QARI, COREQ, Mays and Pope [25]; reviewer developed tools; describe the domains assessed: research team, study design, data analysis and interpretations, reporting). | 6 |
| 12 | Appraisal process | Indicate whether the appraisal was conducted independently by more than one reviewer and if consensus was required. | 6 |
| 13 | Appraisal results | Present results of the quality assessment and indicate which articles, if any, were weighted/excluded based on the assessment and give the rationale. | 6 |
| 14 | Data extraction | Indicate which sections of the primary studies were analysed and how were the data extracted from the primary studies? (e.g. all text under the headings “results /conclusions” were extracted electronically and entered into a computer software). | 5 |
| 15 | Software | State the computer software used, if any | 5 |
| 16 | Number of reviews | Identify who was involved in coding and analysis. | 6 |
| 17 | Coding | Describe the process for coding of data (e.g. line by line coding to search for concepts) | 6 |
| 18 | Study comparison | Describe how were comparisons made within and across studies (e.g. subsequent studies were coded into pre-existing concepts, and new concepts were created when deemed necessary) | 6 |
| 19 | Deviation of themes | Explain whether the process of deriving the themes or constructs was inductive or deductive | 6 |
| 20 | Quotations | Provide quotations from the primary studies to illustrate themes/constructs, and identify whether the quotations were participant quotations of the author’s interpretation. | 7-15 |
| 21 | Synthesis output | Present rich, compelling and useful results that go beyond a summary of the primary studies (e.g. new interpretation, models of evidence, conceptual models, analytical framework, development of a new theory or construct). | 16-17 |

Table S2 Assessment of quality of included studies using the CASP tool

| Author(s)  Alphabetical order | Aims | Method appropriate | Design appropriate | Recruitment appropriate | Data collection  appropriate | Prior experience/  relationship | Ethical | Data Analysis | Clear statement of findings | Value of research |
| --- | --- | --- | --- | --- | --- | --- | --- | --- | --- | --- |
| Abraham et Wilk 2014^22^ | Yes | Yes | Yes | Yes | Yes | No | Unclear | Yes | Yes | Yes |
| Araujo et al 2013^23^ | Yes | Yes | Yes | Yes | Yes | No | Yes | Yes | No | Yes |
| Bandyopadhyay et al 2015^24^ | Yes | Yes | Yes | Yes | Yes | No | Yes | Yes | Yes | Yes |
| Bandyopadhyay et al 2011^25^ | Yes | Yes | Yes | Yes | Yes | No | Yes | Yes | Yes | Yes |
| Carolan 2013^26^ | Yes | Yes | Yes | Yes | Yes | No | Yes | Yes | Yes | Yes |
| Carolan-Olah et al 2017^8^ | Yes | Yes | Yes | Yes | Yes | No | Yes | Yes | Yes | Yes |
| Doran 2008^27^ | Yes | Yes | Yes | Yes | Yes | No | Yes | Yes | Yes | Yes |
| Doran et Davis 2010^28^ | Yes | Yes | Yes | Yes | Unclear | No | Yes | Unclear | Yes | Yes |
| Draffin et al 2016^10^ | Yes | Yes | Yes | Yes | Yes | No | Yes | Yes | Yes | Yes |
| Eades et al 2018^29^ | Yes | Yes | Yes | Yes | Yes | No | Yes | Yes | Yes | Yes |
| Evan et Brien 2005^6^ | Yes | Yes | Yes | Yes | Yes | No | Yes | Yes | Yes | Yes |
| Figueroa Gray 2017^30^ | Yes | Yes | Yes | Yes | Yes | No | Unclear | Yes | Yes | Yes |
| Ge, Albin et al 2016a^31^ | Yes | Yes | Yes | Yes | Yes | No | Yes | Yes | Yes | Yes |
| Ge, Wikby et al 2016b^32^ | Yes | Yes | Yes | Yes | Yes | No | Yes | Yes | Yes | Yes |
| Han et al 2015^33^ | Yes | Yes | Yes | Yes | Yes | No | Yes | Yes | Yes | Yes |
| Hirst et al 2012^34^ | Yes | Yes | Yes | Yes | Yes | No | Yes | Yes | Yes | Yes |
| Hjelm et al 2005^38^ | Yes | Yes | Yes | Yes | Yes | No | Yes | Yes | Yes | Yes |
| Hjelm et al 2008^40^ | Yes | Yes | Yes | Yes | Yes | No | Yes | Yes | Yes | Yes |
| Hjelm et al 2009^37^ | Yes | Yes | Yes | Yes | Yes | No | Yes | Yes | Yes | Yes |
| Hjelm et al 2018^36^ | Yes | Yes | Yes | Yes | Yes | No | Yes | Yes | Yes | Yes |
| Hjelm et al 2012a^39^ | Yes | Yes | Yes | Yes | Yes | No | Yes | Yes | Yes | Yes |
| Hjelm et al 2012b^35^ | Yes | Yes | Yes | Yes | Yes | No | Yes | Yes | Yes | Yes |
| Hui et al 2014^41^ | Yes | Yes | No | Yes | Yes | No | Yes | Yes | Yes | Yes |
| Kaptein et al 2015^42^ | Yes | Yes | Yes | Yes | Yes | No | Yes | Yes | Yes | Yes |
| Kilgour et al 2015^43^ | Yes | Yes | Yes | Yes | Yes | No | Yes | Yes | Yes | Yes |
| Lawson et Rajaram 1994^44^ | No | Unclear | No | Yes | No | No | No | No | No | No |
| Lie et al 2013^45^ | Yes | Yes | Yes | Yes | Yes | No | Yes | Yes | Yes | Yes |
| Neufeld 2011^46^ | Yes | Yes | Yes | Yes | Yes | No | Yes | Yes | Yes | Yes |
| Nieslen et al 2015^47^ | Yes | Yes | Yes | Yes | Yes | No | Yes | Yes | Yes | Yes |
| Parsons 2018^48^ | Yes | Yes | Yes | Yes | Yes | Yes | Yes | Yes | Yes | Yes |
| Pennington et al 2017^49^ | Yes | Yes | Yes | Yes | Yes | No | Yes | Yes | Yes | Yes |
| Persson et al 2010^9^ | Yes | Yes | Yes | Yes | Yes | No | Yes | Yes | Yes | Yes |
| Rafii et al 2017^50^ | Yes | Yes | Yes | Yes | Yes | No | Yes | Yes | Yes | Yes |
| Razee et al 2010^51^ | Yes | Yes | Yes | Yes | Yes | No | Yes | Yes | Yes | Yes |
| Salomon et Soares 2004^52^ | Yes | Yes | No | Unclear | Yes | No | Yes | Unclear | No | No |
| Svensson et al 2018^53^ | Yes | Yes | No | Yes | No | No | Yes | Yes | Yes | Yes |
| Tang et al 2015^54^ | Yes | Yes | Yes | Yes | Yes | No | Yes | Yes | Yes | Yes |
| Tierney et al 2015^55^ | Yes | Yes | Yes | Yes | Yes | No | Yes | Yes | Yes | Yes |
| Trutnovsky 2012^56^ | Yes | Yes | Yes | Unclear | No | No | Yes | No | No | No |
| Wah et al 2018^57^ | Yes | Yes | No | Yes | Yes | No | Yes | Yes | Yes | Yes |
| Whitty-Rodgers et al 2016^58^ | Yes | Yes | Yes | Yes | Yes | Yes | Yes | Yes | Yes | Yes |
